# Intensive care management of a patient with necrotizing fasciitis due to non-O1/O139 *Vibrio cholerae* after traveling to Taiwan: a case report

**DOI:** 10.1186/s12879-020-05343-6

**Published:** 2020-08-24

**Authors:** Keisuke Tsuruta, Toru Ueyama, Tomoo Watanabe, Kenichi Nakano, Kenji Uno, Hidetada Fukushima

**Affiliations:** 1 Emergency Department, Minaminara General Hospital, 8-1 Ooazafukugami, Ooyodocho, Yoshino-gun, Nara, 638-0833 Japan; 2 Orthopedic Department, Minaminara General Hospital, Nara, Japan; 3 Infectious Diseases Department, Minaminara General Hospital, Nara, Japan; 4 Department of Emergency and Critical Care Medicine, Nara Medical University, Nara, Japan

**Keywords:** Necrotizing fasciitis, *Vibrio cholerae*, Taiwan, Massage, Septic shock, Polymyxin B

## Abstract

**Background:**

*Vibrio cholerae* are oxidase-positive bacteria that are classified into various serotypes based on the O surface antigen. *V. cholerae* serotypes are divided into two main groups: the O1 and O139 group and the non-O1/non-O139 group. O1 and O139 *V. cholerae* are related to cholera infection, whereas non-O1/non-O139 *V. cholerae* (NOVC) can cause cholera-like diarrhea. A PubMed search revealed that only 16 cases of necrotizing fasciitis caused by NOVC have been recorded in the scientific literature to date. We report the case of a Japanese woman who developed necrotizing fasciitis caused by NOVC after traveling to Taiwan and returning to Japan.

**Case presentation:**

A 63-year-old woman visited our hospital because she had experienced left knee pain for the past 3 days. She had a history of colon cancer (Stage IV: T3N3 M1a) and had received chemotherapy. She had visited Taiwan 5 days previously, where she had received a massage. She was diagnosed with septic shock owing to necrotizing fasciitis. She underwent fasciotomy and received intensive care. She recovered from the septic shock; however, after 3 weeks, she required an above-knee amputation for necrosis and infection. Her condition improved, and she was discharged after 22 weeks in the hospital.

**Conclusions:**

With the increase in tourism, it is important for clinicians to check patients’ travel history. Clinicians should be alert to the possibility of necrotizing fasciitis in patients with risk factors. Necrotizing fasciitis caused by NOVC is severe and requires early fasciotomy and debridement followed by intensive postoperative care.

## Background

*Vibrio cholerae* are curved gram-negative rod (GNR) bacteria that are oxidase positive. They are classified into various serotypes based on the O surface antigen. *V. cholerae* serotypes are divided into two main groups: the O1 and O139 group and the non-O1/non-O139 group [1]. O1 and O139 *V. cholerae* are related to cholera infection, whereas non-O1/non-O139 *V. cholerae* (NOVC) can cause cholera-like diarrhea. NOVC are found as autochthonous microbes in coastal and marine environments [2]. Outbreaks of cholera-like illness caused by NOVC have been reported in the United States (O141 and O75), former Czechoslovakia (O37), Sudan (O37), Peru (O10, O12), and Mexico (O14) [3–5]. Moreover, NOVC can cause a range of extraintestinal infections, including bacteremia, meningitis, pneumonia, peritonitis, cholangitis, salpingitis, and soft-tissue infection [6]. Seafood, including oysters, fishes, shrimps, clams, mussels, and apple snail, is the most common source of infection (53.9%) [7]. A PubMed search revealed that only 16 cases of necrotizing fasciitis caused by NOVC have been reported in the scientific literature to date. We report the case of a patient who developed necrotizing fasciitis and septic shock caused by NOVC, which necessitated an above-knee amputation of her left leg.

## Case presentation

**Fig. 1:**
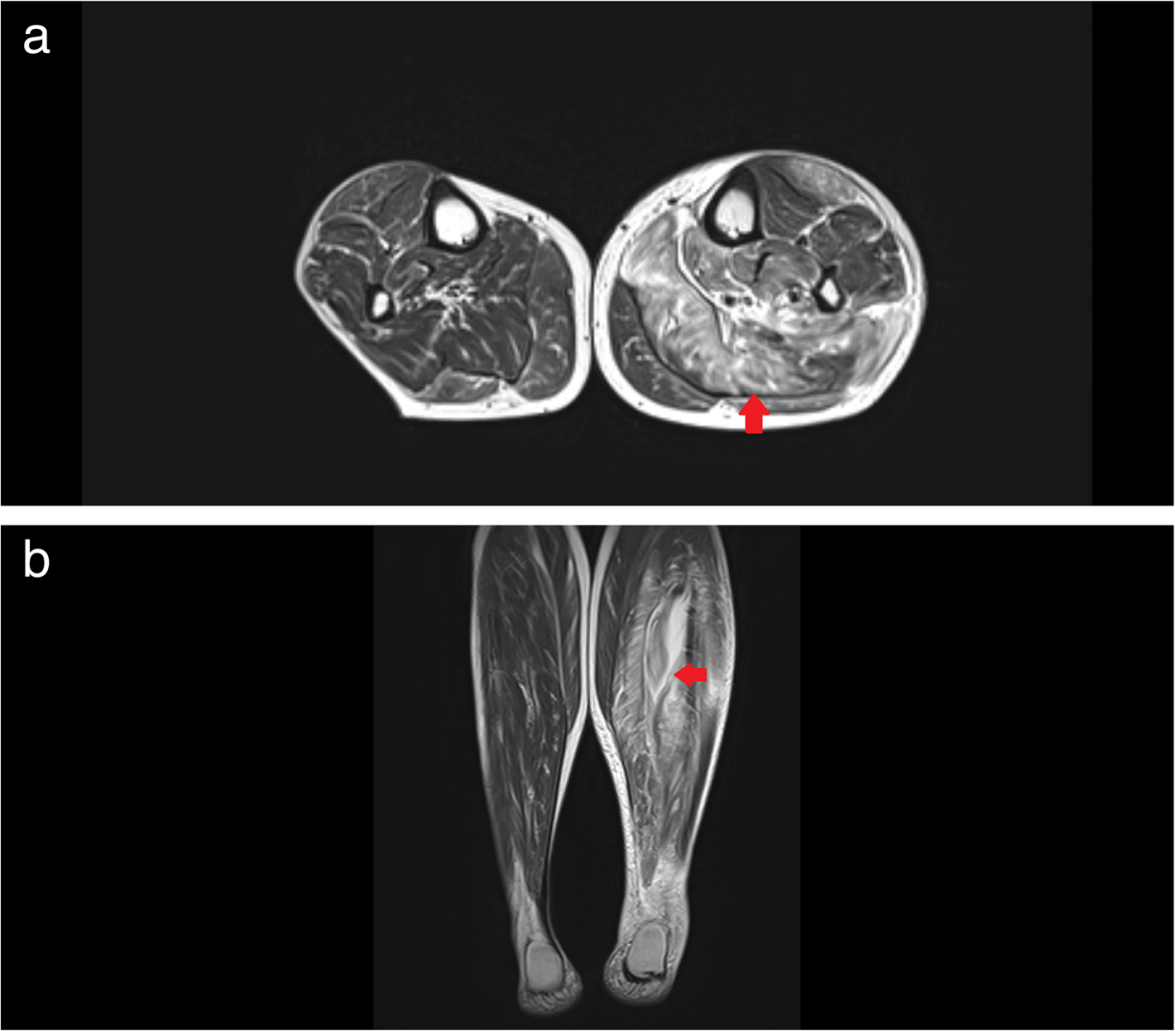
T2-weighted magnetic resonance images of the patient’s lower legs. **a**. Coronal image; **b**. Axial image. These images show that the soleus and posterior tibial muscles on the left lower leg (indicated by red arrows) are swollen and inflamed

A 63-year-old woman visited Minaminara General Hospital in Nara, Japan, because she had experienced left knee pain for 3 days prior to her visit. She had been diagnosed with colon cancer (Stage IV: T3N3 M1a) 2 years and 5 months previously and had undergone surgery and received chemotherapy. Her most recent dose of chemotherapy was administered 20 days before her initial consultation. She had visited Taiwan 5 days previously, where she had received a massage. After the massage, she developed gradually worsening pain in her lower left leg. On presentation, she was able to walk unaided, and she reported her history of colon cancer and recent travel. As we suspected that the pain in her leg could be due to necrotizing fasciitis, we requested magnetic resonance imaging (MRI) of her left lower leg. The images showed a swollen soleus muscle and posterior tibial muscle, and the T2-weighted image showed hyperintensity of the muscle tissue (Fig. 1). After the MRI, our patient’s condition deteriorated and the following vital signs were observed: blood pressure (BP), 89/50 mmHg; heart rate, 101 beats/min; respiratory rate, 18 breaths/min; and temperature, 36.3 °C. The results of arterial blood gas analysis were as follows: pH, 7.4; PaCO_2_, 26.7 mmHg; HCO_3_^−^, 18.8 mmHg; base excess (BE), − 6.9 mEq/L; and lactate, 3.20 mmol/L. The patient’s laboratory test results were as follows: C-reactive protein (CRP), 42.83 mg/dL; blood urea nitrogen (BUN), 73.3 mg/dL; creatinine, 1.78 mg/dL; procalcitonin, 16.06 ng/mL; N-terminal pro-brain natriuretic peptide (Nt-proBNP), 29,506 pg/mL; and fibrin/fibrinogen degradation products (FDP), 16.7 μg/mL.

**Fig. 2:**
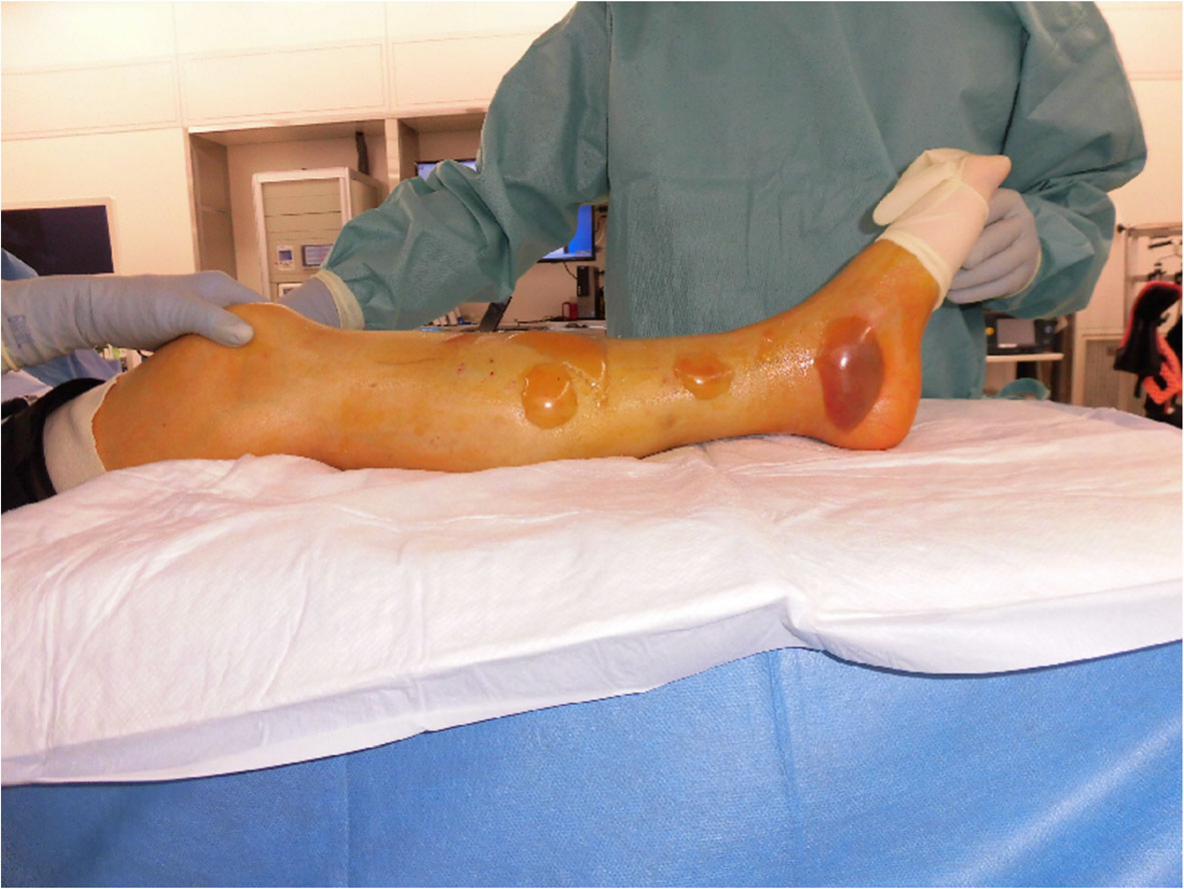
Photographs of lesions in the patient’s leg. The patient’s leg before surgery shows multiple large blisters

**Fig. 3:**
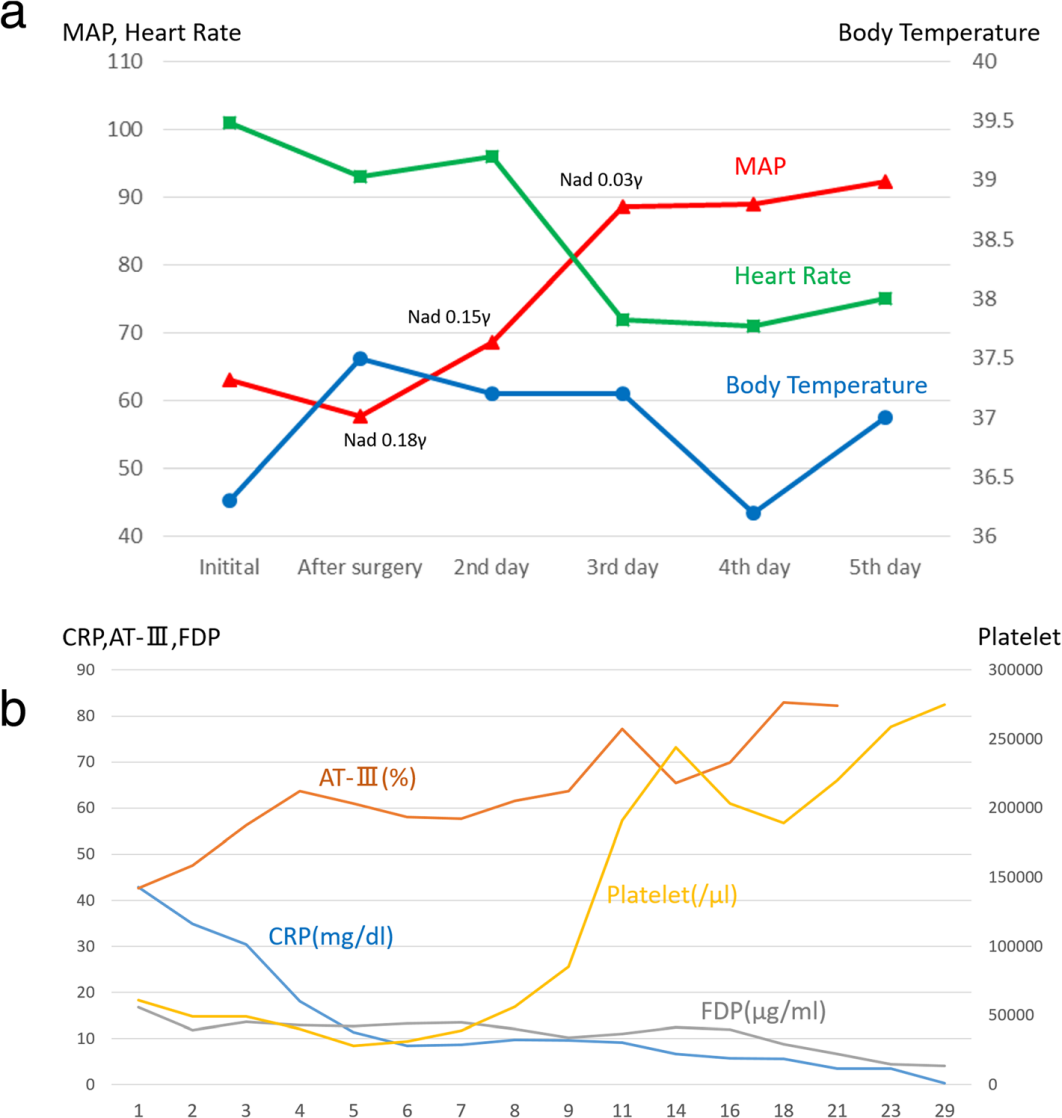
Change of vital signs and laboratory data during the hospital admission. **a**. Changes in the patient’s vital signs during days 0–5 of hospitalization. **b**. Changes in patient’s blood biochemistry during days 1–29 of hospitalization. (AT-III, Antithrombin III; CRP, C-reactive protein; FDP, fibrin/fibrinogen degradation products; MAP, mean arterial pressure; Nad, noradrenaline)

Intravenous infusion of meropenem and noradrenaline was initiated, and the patient underwent emergency surgery. Before the surgery, the compartment pressure of her left leg was measured by simple needle manometry. The pressures were as follows: 63 mmHg, 26 mmHg, 32 mmHg, and 32 mmHg in the anterior, lateral, superficial posterior, and deep posterior compartments, respectively. Some muscle tissues in the anterior and deep posterior compartments were necrotic. For double incision fasciotomy, a relaxation incision was made on her left knee [8], and the affected area was irrigated and debrided (Fig. 2). After the surgery, her blood pressure was low, and therefore, we administered polymyxin B direct hemoperfusion (PMX-DHP), to trap endotoxins, and continuous veno-venous hemodiafiltration (using HEMOFEEL CH-1.3 W, Toray Medical Co., Ltd., Urayasu, Japan). As a slightly curved GNR that was oxidase positive was detected in her blood, we diagnosed her with necrotizing fasciitis and septic shock caused by *Vibrio* species. We changed the antibiotics from meropenem to ceftriaxone, levofloxacin, and minocycline. We used the PMX-DHP once again and tapered the dose of noradrenalin gradually. We discontinued noradrenalin on Day 4 postoperatively. On Day 6 postoperatively, the organism was identified as NOVC. The susceptibility of antibiotics was confirmed postoperatively on Day 12, and we discontinued levofloxacin (Table 1). Although the patient’s general condition improved, there was a discharge of pus from the postoperative wound. On Day 14 postoperatively, a second debridement was performed. Several muscles in the patient’s left leg, including the anterior tibial muscle, had become necrotic, and the necrosis had spread to her knee. On Day 21 postoperatively, an above-knee amputation was performed. Her vital signs and laboratory data obtained since admission are shown in Fig. 3. Her condition improved and she was discharged 22 weeks after admission.

**Table 1:** Susceptibility of antibiotics

Antibiotics	Minimal inhibitory concentration
Ampicillin	S 2
Piperacillin	S 16
Ceftazidime	S 4
Imipenem/Cilastatin	S ≦1
Amoxicillin/Clavulanate	S 4
Gentamicin	S 2
Minocycline	S ≦1
Chloramphenicol	S ≦2
Sulfamethoxazole-Trimethoprim	S ≦10
Levofloxacin	S ≦0.5
Fosfomycin	R >128

## Discussion and conclusion

Sixteen cases of necrotizing fasciitis caused by NOVC have been previously reported (Table 2) [9–17]. The majority of patients were exposed to seawater or had an injury. In rare cases, vigorous massage is one of the risk factors of necrotizing fasciitis [18]. However, the patient in the present case had a risk of NOVC infection because of colon cancer and immunosuppression due to chemotherapy as she received chemotherapy within a month. Thus, in this case, the source of the NOVC remains unknown. As the patient did not report any exposure to sea water or eating seafood, the only potential cause of injury to her left leg was the massage she received. Therefore, we speculate that the massage might have been the source of the NOVC, based on the circumstantial evidence. We administered blood purification therapy using PMX-DHP and veno-venous hemodiafiltration for septic shock. Although no previous studies have reported the use of PMX-DHP for NOVC, a study reported the use of PMX for *V. vulnificus* [19]. Third-generation cephalosporins, tetracycline, and fluoroquinolone were used for severe *Vibrio* infections. Tetracycline combined with the fluoroquinolone or a parenteral third-generation cephalosporin followed by oral fluoroquinolones or doxycycline was recommended for invasive NOVC infections [10, 14]. An in vitro study revealed that cefotaxime and minocycline have a synergistic effect in the treatment for *V. cholerae* infections [20]. As patients with NOVC bacteremia require antibiotic treatment for at least 1 month [14], we administered ceftriaxone and minocycline for 1 month. Necrotizing soft-tissue infections caused by NOVC are more lethal than those caused by *V. vulnificus* [21].

**Table 2:** Clinical characteristics of patients with non-O *Vibrio cholerae* necrotizing fasciitis

Year of report (Source)	Age, Sex	Underlying diseases/risk factors	Treatment	Outcome	Country	O-Antigen	Epidemiologic exposure
1995 (9)	51 M	Diabetes mellitus	Surgery (amputation, multiple debridement),and antibiotics (ticarcillin/clavulanate, imipenem, gentamicin, clindamycin)	Survived	USA		Exposure of a chronic plantar ulcer to sand in a bathhouse
1998 (10)	48 M	Cirrhosis	Surgery, cefotaxime, minocycline, cefotaxime	Survived	Taiwan		
1998 (10)	79 F	Cirrhosis, congestive heart failure	Surgery, ceftriaxone	Died	Taiwan		
2004 (11)	68 M	Cirrhosis, diabetes mellitus	Surgical debridement, ceftazidime, doxycycline	Died	Taiwan	O56	
2004 (12)	70 M	Hepatitis C	Surgery, antibiotics (third-generation cephalosporin, doxycycline)	Died	Taiwan		Handling seafood
2004 (12)	63 M	Hepatitis, steroids	Surgery, antibiotics (third-generation cephalosporin, doxycycline)	Survived	Taiwan		Exposure to sea water
2006 (13)	52 M	Cirrhosis	Surgery, clindamycin, ceftazidime, tetracycline	Survived	Taiwan		Probable wound infection
2009 (14)	57 M	Cirrhosis, hepatitis C, diabetes mellitus	Surgery, antibiotics	Died	Taiwan		Consumption of raw seafood
2009 (14)	58 M	Cirrhosis, hepatitis B, hepatitis C, diabetes mellitus	Surgery, antibiotics	Died	Taiwan		Seawater exposure
2009 (14)	69 M	Cirrhosis, diabetes mellitus	Surgery, antibiotics	Died	Taiwan		Seawater exposure
2009 (14)	70 M	Cirrhosis	Surgery, antibiotics	Survived	Taiwan		Seawater exposure
2009 (14)	62 M	COPD	Surgery, antibiotics	Survived	Taiwan		Insect bite wound infection
2011 (15)	49 M	HIV, hepatitis C, cirrhosis	Surgical debridement, daptomycin, levofloxacin	Survived	Italy	O137	Minor abrasion exposed to seawater
2016 (16)	73 M	Diabetes mellitus	Surgical debridement, piperacillin/tazobactam, fosfomycin	Survived	Austria		Seawater exposure
2016 (16)	80 M	Ichthyosis, cellulitis	Surgical debridement, piperacillin/tazobactam, tigecycline, metronidazole	Died	Austria		Seawater exposure
2016 (17)	6 3 M	None	Surgical debridement, penicillin, gentamicin, metronidazole	Survived	Croatia	O8	Seawater exposure

*COPD* chronic constructive pulmonary disease

To conclude, we treated a woman with necrotizing fasciitis and septic shock caused by NOVC. This case illustrates that early fasciotomy and debridement are necessary for severe necrotizing fasciitis caused by NOVC, and prolonged intensive care may be required after surgery.

## Data Availability

All data are included in this published article.

## Abbreviations

BUN: Blood urea nitrogen
FDP: Fibrinogen degradation products
GNR: Gram-negative rod
MRI: Magnetic resonance imaging
NOVC: Non-O1/O139 *Vibrio cholerae*
Nt-proBNP: N-terminal pro-brain natriuretic peptide
PMX-DHP: Polymyxin B direct hemoperfusion
